# Metachronous Bilateral Nephrectomy, Supratrigonal Cystectomy, and Living Donor Renal Transplantation in a Patient With Chronic Kidney Disease due to Interstitial Cystitis and Total Urethral Stricture

**DOI:** 10.1155/carm/3103124

**Published:** 2025-08-25

**Authors:** Rashad Sholan, Rufat Aliyev, Anar Almazkhanli, Jalal Gasimov, Nargiz Bakhshaliyeva, Malahat Sultan

**Affiliations:** ^1^Scientific Research Center, State Security Service Military Hospital, Baku, Azerbaijan; ^2^Scientific Research Center, Azerbaijan Medical University, Baku, Azerbaijan; ^3^Department of Kidney Diseases and Organ Transplantation, State Security Service Military Hospital, Baku, Azerbaijan; ^4^Department of the Emergency Medicine, State Security Service Military Hospital, Baku, Azerbaijan; ^5^Department of Radiology, Azerbaijan Medical University, Baku, Azerbaijan

**Keywords:** Bricker ileal conduit, interstitial cystitis, renal transplantation, supratrigonal cystectomy

## Abstract

**Introduction:** Interstitial cystitis (IC) is a chronic pelvic pain syndrome characterized by urinary urgency, frequency, and pain. Although the exact cause of IC is unclear, severe cases may lead to chronic kidney disease (CKD), requiring complex surgical interventions.

**Case Presentation:** This case report presents a 46-year-old male with IC complicated by CKD secondary to total urethral stricture. Following recurrent surgeries for urinary tract infections and strictures, the patient underwent a staged surgical approach involving bilateral nephrectomy, supratrigonal cystectomy, and renal transplantation with Bricker ileal conduit diversion. Postoperatively, renal function was fully restored, and IC-related symptoms resolved.

**Conclusion:** This case underscores the efficacy of major surgical interventions for refractory IC, highlighting the need for individualized, multidisciplinary management in complex cases.

**Summary**

• This case report highlights a novel surgical approach for interstitial cystitis (IC) with chronic kidney disease (CKD), combining bilateral nephrectomy, supratrigonal cystectomy, and kidney transplantation.
• By removing infection sources and restoring urinary function, this staged surgery significantly improved quality of life for a patient unresponsive to other treatments, suggesting major surgery as a viable option for selected IC patients with severe, persistent symptoms.

## 1. Introduction

IC is a chronic pelvic pain syndrome marked by urinary urgency, frequency, pelvic tenderness, pain with bladder filling, and relief after voiding [1]. Women are significantly more likely to develop IC than men, with a female-to-male ratio of 9 to 1 [2]. The exact cause of IC is unknown, but studies suggest potential contributing factors, including altered HLA Class I and II antigen expression in the bladder epithelium, decreased uroplakin and chondroitin sulfate levels, changes in the glycosaminoglycan layer, Tamm–Horsfall protein defects, and increased IL-6 and P2X3 ATP receptor expression [3–5]. A common complaint among IC patients is the constant urge to urinate in order to maintain a low bladder volume, which helps minimize pain [6, 7]. Without appropriate treatment, the quality of life for these patients is severely impacted.

The primary goals of IC treatment are to improve the patient's quality of life, reduce urinary urgency and frequency, and relieve bladder pain. However, treatment options vary widely due to the heterogeneity of the disease and the unclear pathogenesis. Major surgical interventions are typically considered a last resort. Three independent factors have been associated with better prognoses after major surgery: the presence of Hunner lesions (HL), reduced bladder capacity, and extensive bladder fibrosis [8]. Studies have demonstrated that patients who undergo major surgeries, such as supratrigonal cystectomy and augmentation cystoplasty using ileum or ileocecum, report high satisfaction with long-term outcomes. For patients with IC complicated by CKD, renal transplantation is also an option [9]. This case report presents a complex therapeutic approach involving supratrigonal cystectomy, bilateral nephrectomy, and living donor renal transplantation, highlighting the surgical challenges and clinical outcomes in a patient with CKD secondary to IC and total urethral stricture.

## 2. Case Report

A 46-year-old male patient presented to our Emergency Department with complaints of intense pelvic discomfort, dysuria, flank pain, difficulty urinating, and 40°C fever. His medical history included five previous surgical interventions for severe urinary tract infections and iatrogenic urethral stricture. The patient reported undergoing continuous urinary catheterization and urethral balloon dilation. Additionally, the patient had a history of Type 2 diabetes mellitus and CKD (with urine output and no history of hemodialysis). His body mass index was 31 kg/m^2^.

On physical examination, significant suprapubic and bilateral costovertebral angle tenderness were noted. Blood tests revealed the following: white blood cell count of 19,000/µL, c-reactive protein of 110 mg/L, creatinine level of 3.29 mg/dL, hemoglobin of 12.4 g/dL, and estimated glomerular filtration rate of 20.4 mL/min/1.73 m^2^. Urinalysis indicated the presence of an infection, and a computed tomography scan revealed bilateral atrophic kidneys. The patient was diagnosed with an acute bacterial infection, urinary tract infection, sepsis, and postrenal CKD. He was admitted to the urology department for further management.

During follow-up, the patient was treated with antibiotics. Once the infection subsided, a cystourethroscopy was performed. Findings included Stage 4 CKD, hemorrhagic bladder ulcers, and a long-standing urethral stricture. Bladder capacity was measured at 30 cc. A bladder biopsy revealed collagen deposition and chronic inflammatory mast cell proliferation. Bilateral nephrectomy and supratrigonal cystectomy were performed to eliminate infection foci.

**Figure 1:**
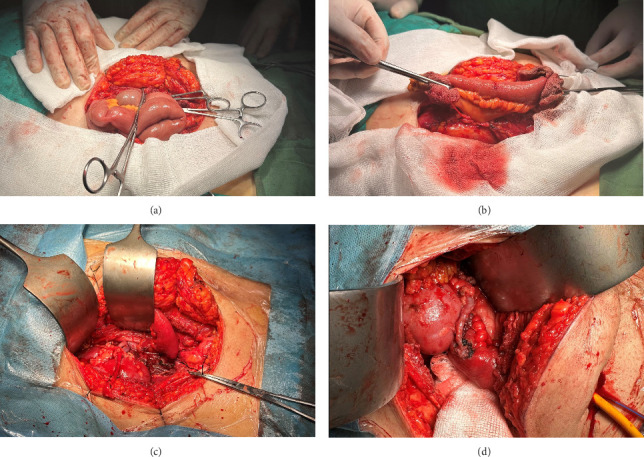
Bricker ileal conduit diversion procedure. The abdomen was accessed through a right modified Gibson incision, and the peritoneum was opened. Starting 20-cm proximal to the ileocecal valve, a 20-cm section of the ileum was removed (a). After being resected, the section was extraperitoneally implanted in its anatomic location while maintaining its mesentery (b). The renal vein and artery were anastomosed to the external iliac vein and artery, respectively (c). The ureter was anastomosed to the iliac loop (d).

One month later, the patient underwent a living donor kidney transplant and a Bricker ileal conduit diversion. During the procedure, a right modified Gibson incision was made to expose the right iliac artery and vein. A 20-cm segment of the terminal ileum, with an intact blood supply, was isolated to create a Bricker loop. The peritoneum was closed, and the Bricker loop was placed in the extraperitoneal region. The renal vein and artery were anastomosed to the external iliac vein and artery, respectively, followed by a ureteroileal anastomosis. The warm ischemia time was 54 s, and the cold ischemia time was 95 min (Figure 1).

The patient was treated with trimethoprim/sulfamethoxazole prophylaxis for 3 months. No short-term or long-term postoperative complications were observed during this period. At the 3-month follow-up postsurgery, the patient's creatinine level was 0.8 mg/dL.

## 3. Discussion

Due to the paucity of evidence regarding the pathophysiology of IC, most current treatments address only symptom management [10]. A number of behavioral, nutritional, pharmacological, intravesical, and surgical treatments have been suggested; however, the effectiveness of these therapies is still lacking [11].

Guidelines addresses the use of major surgery—such as substitution cystoplasty or urinary diversion with or without cystectomy—in carefully selected patients with severe bladder-centric symptoms or in cases of end-stage small fibrotic bladder [1]. These surgeries are considered when all other therapies have failed to alleviate symptoms and improve quality of life. While some patients experience complete or near-complete symptom relief, others may suffer from persistent pain, even after bladder removal, or develop new complications. The evidence supporting these procedures is limited due to small patient cohorts, variation in surgical techniques, and inconsistent outcome reporting, making it difficult to weigh the benefits against the risks and burdens accurately [11, 12]. Careful patient selection is crucial for successful outcomes in major bladder surgery. Surgery should be reserved for those who have exhausted all other treatment options and whose symptoms are clearly bladder-related. Key predictors of success include the presence of an end-stage fibrotic bladder, small bladder capacity under anesthesia, and the presence of HL [13, 14]. A thorough preoperative evaluation is necessary to rule out pain sources outside the bladder, and multidisciplinary assessments are recommended to ensure surgery is appropriate.

The choice of a surgical method is usually influenced by surgeon and patient-specific factors. Surgical options include supratrigonal cystectomy with augmentation and supravesical urinary diversion. Our case represents the first reported instance in the literature where bilateral nephrectomy, supratrigonal cystectomy, and living donor renal transplantation were performed in a patient with IC. The most notable aspect of this case was the extensive damage to all organs of the urinary system. Through staged surgical intervention, the infection foci were eliminated, and renal transplantation successfully restored urinary function. The patient's IC-related symptoms were completely resolved.

## 4. Conclusion

This case represents the first reported instance of bilateral nephrectomy, supratrigonal cystectomy, and living donor renal transplantation in a patient with IC and CKD. Staged surgery successfully eliminated infection foci, restored urinary function, and resolved IC-related symptoms. Major surgery, while complex, can provide significant symptom relief and improved quality of life in carefully selected patients.

## Data Availability

Data sharing is not applicable to this article as no new data were created or analyzed in this study.

## References

[1] Clemens J. Q., Erickson D. R., Varela N. P., Lai H. H. (2022). Diagnosis and Treatment of Interstitial Cystitis/Bladder Pain Syndrome. *The Journal of Urology*.

[2] Simon L. J., Landis J. R., Erickson D. R., Nyberg L. M. (1997). The Interstitial Cystitis Data Base Study: Concepts and Preliminary Baseline Descriptive Statistics. *Urology*.

[3] Slobodov G., Feloney M., Gran C., Kyker K. D., Hurst R. E., Culkin D. J. (2004). Abnormal Expression of Molecular Markers for Bladder Impermeability and Differentiation in the Urothelium of Patients With Interstitial Cystitis. *The Journal of Urology*.

[4] Graham E., Chai T. C. (2006). Dysfunction of Bladder Urothelium and Bladder Urothelial Cells in Interstitial Cystitis. *Current Urology Reports*.

[5] Hurst R. E., Moldwin R. M., Mulholland S. G. (2007). Bladder Defense Molecules, Urothelial Differentiation, Urinary Biomarkers, and Interstitial Cystitis. *Urology*.

[6] Tsai C. F., Ouyang W. C., Tsai S. J., Hong C. J., Lin T. L. (2010). Risk Factors for Poor Sleep Quality Among Patients With Interstitial Cystitis in Taiwan. *Neurourology and Urodynamics*.

[7] Lai H. H., Vetter J., Jain S., Gereau R. W., Andriole G. L. (2014). The Overlap and Distinction of Self-Reported Symptoms Between Interstitial Cystitis/Bladder Pain Syndrome and Overactive Bladder: A Questionnaire Based Analysis. *The Journal of Urology*.

[8] Whitmore K. E., Fall M., Sengiku A., Tomoe H., Logadottir Y., Kim Y. H. (2019). Hunner Lesion Versus Non-Hunner Lesion Interstitial Cystitis/Bladder Pain Syndrome. *International Journal of Urology*.

[9] Li J., Yi X., Ai J. (2022). Broaden Horizons: The Advancement of Interstitial Cystitis/Bladder Pain Syndrome. *International Journal of Molecular Sciences*.

[10] Garzon S., Laganà A. S., Casarin J. (2020). An Update on Treatment Options for Interstitial Cystitis. *Przegląd Menopauzalny*.

[11] Kim H. J., Lee J. S., Cho W. J. (2014). Efficacy and Safety of Augmentation Ileocystoplasty Combined With Supratrigonal Cystectomy for the Treatment of Refractory Bladder Pain Syndrome/Interstitial Cystitis With Hunner’s Lesion. *International Journal of Urology*.

[12] Mateu Arrom L., Gutiérrez Ruiz C., Mayordomo Ferrer O., Martínez Barea V., Palou Redorta J., Errando Smet C. (2019). Long-Term follow-Up After Cystectomy for Bladder Pain Syndrome: Pain Status, Sexual Function and Quality of Life. *World Journal of Urology*.

[13] Redmond E. J., Flood H. D. (2017). The Role of Reconstructive Surgery in Patients With End-Stage Interstitial Cystitis/Bladder Pain Syndrome: Is Cystectomy Necessary?. *International Urogynecology Journal*.

[14] Andersen A. V., Granlund P., Schultz A., Talseth T., Hedlund H., Frich L. (2012). Longterm Experience With Surgical Treatment of Selected Patients With Bladder Pain Syndrome/Interstitial Cystitis. *Scandinavian Journal of Urology and Nephrology*.

